# Novel Missense DNA Variants in the IL2RG Gene Identified in Slovak X-linked Severe Combined Immunodeficiency Disease Patients: A Case Report

**DOI:** 10.7759/cureus.75872

**Published:** 2024-12-17

**Authors:** Gabriela Krasnanska, Gabriela Blandova, Marian Baldovic, Maria Andrejkova, Michal Konecny

**Affiliations:** 1 Department of Biology, Institute of Biology and Biotechnology, Faculty of Natural Sciences, University of St. Cyril and Methodius, Trnava, SVK; 2 Laboratory of Genomic Medicine, GHC GENETICS SK, Comenius University Science Park, Bratislava, SVK; 3 Department of Paediatrics, Faculty of Medicine, Pavol Jozef Safarik University and Children's Faculty Hospital, Centre for Inborn Errors of Immunity and Clinical Genomics, Kosice, SVK

**Keywords:** il2rg gene, inborn errors of immunity, interleukin 2 gamma receptor, wes, x-scid

## Abstract

X-linked severe combined immunodeficiency disease (X-SCID) is a form of inborn errors of immunity (IEI) associated with causal DNA variants of the *IL2RG* gene. Patients with X-SCID are characterized by a combination of cellular and humoral immunodeficiencies associated with increased susceptibility to infections. The presented cases constituted two unrelated male patients from the Slovak population. Proband A was primarily hospitalized at the age of three months because of recurrent fever, vomiting, and lethargy, and the atypical immunophenotype was determined to be T-B-NK-. For proband B, the first hospitalization occurred at the age of eight months because of generalized impetiginized dermatitis. Whole exome sequencing (WES) was performed via a comprehensive approach in patients with undefined IEI, and causal DNA variants were confirmed by Sanger sequencing. WES analysis in probands identified the currently undescribed hemizygous variants p.Asn84Thr and p.Val213Ala in the *IL2RG* gene. Segregation analysis of p.Asn84Thr indicated a de novo origin, and p.Val213Ala was detected only in the asymptomatic proband's mother. We comprehensively reconsidered and scored both variants based on biological and clinical aspects. Finally, taking all the information into account, we classified p.Asn84Thr as likely pathogenic and p.Val213Ala as likely pathogenic with mild penetrance based on the fulfilled ACMG (American College of Medical Genetics and Genomics) criteria for computational predictions, clinical correlations, localization at functional site, and de novo status. With the WES approach, we identified two novel, not yet reported, *IL2RG* variants in the Slovak population of X-SCID patients. These findings strengthen the fact that rapid and comprehensive molecular-genetic diagnostics of IEI is necessary for the early definition of precise diagnosis, which further enables appropriate treatment and patient management.

## Introduction

Severe combined immunodeficiency disease (SCID) represents a subgroup of inborn errors of immunity (IEI) with an extremely low prevalence of 1:40,000-75,000. DNA defects associated with SCID are inherited predominantly in a recessive pattern. The most common form of SCID is X-SCID, which is recessively inherited X and affects approximately 46% of SCID patients [1]. X-SCID is a disorder of adaptive immunity caused by mutations in the IL2 receptor common gamma chain (*IL2RG*) gene and is typically associated with a T-B+NK- immunophenotype. Nevertheless, hypomorphic mutations of the *IL2RG *gene have been described in the literature, leading to X-SCID with a milder phenotype, also known as X-CID [2].

The *IL2RG *gene is located on Xq13.1 and spans 4.5 kb of gDNA, which corresponds to 1,560 bp of cDNA. The gene is structured into eight exons and encodes a protein consisting of 369 amino acids, with a molecular weight of 42.3 kDa comprising four functional domains (UniProt ID: P31785). This protein is known as the interleukin 2 common gamma chain receptor (IL2RG), also known as γC, or CD132 [3]. IL2RG represents a crucial transmembrane signaling component among the family of receptors known as interleukin receptors and various cytokines, including IL2, IL4, IL7, IL9, IL15, and IL21, that transmit signals through this receptor subunit. Upon cytokine-receptor binding, three main signaling pathways that facilitate cellular differentiation and proliferation are activated: the PI3K-AKT, RAS-MAPK, and JAK-STAT pathways [4].

Various causal DNA variants in the *IL2RG *gene may cause different phenotypes of X-SCID, accounting for approximately 350 causal DNA variants. Patients with defective IL2RG protein present increased susceptibility to bacterial, viral, and fungal infections and additional features, such as frequent pneumonia, chronic diarrhea, and a poor health state in the early phase of disease onset. These symptoms are characteristic of the typical form of X-SCID and are usually lethal due to opportunistic infections unless patients are treated with allogeneic hematopoietic stem cell transplantation or gene therapy [5,6]. However, patients may also present a milder form of X-SCID, which is associated with leaky or hypomorphic DNA variants and is characterized by normal to moderately reduced T and NK cell numbers and decreased function [7]. Hypomorphic DNA variants may lead to the generation of partially functional proteins and are usually studied by functional assays and computational methods, which predict their impact on protein function. The combination of these approaches closely clarifies their influence on the clinical phenotypes of patients.

Actually, there were not any published results from the Slovak population regarding molecular-genetic analysis of X-SCID. Generally, *IL2RG* causal variants are rarely detected worldwide and even the de novo origin of these mutations is an uncommon event. Here, we report a pilot Slovak study of *IL2RG* causal DNA variants in patients with X-SCID disease and the comprehensive description and reclassification data of two novel, currently unreported DNA variants.

## Case presentation

**Figure 1:**
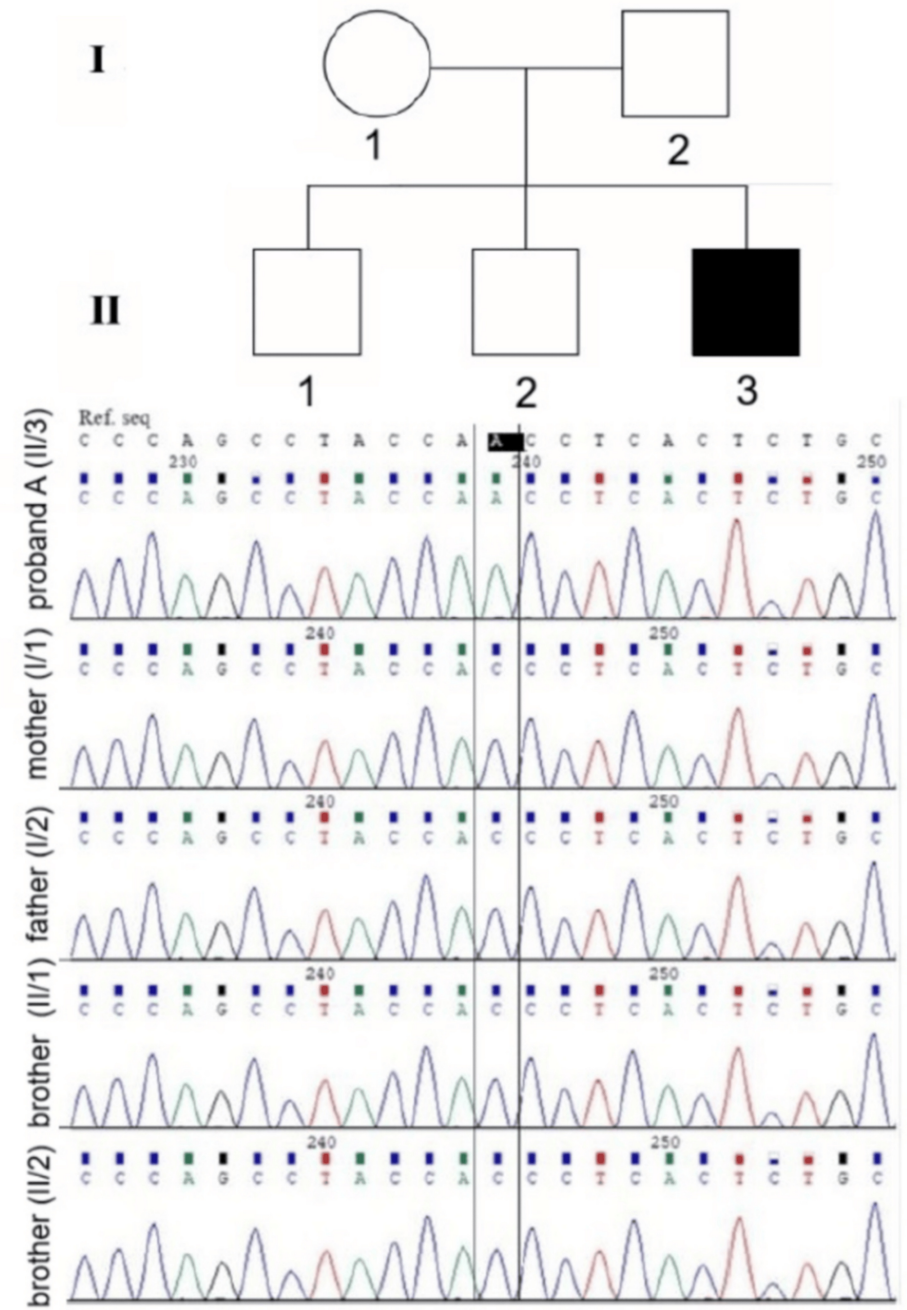
Pedigree and Sanger sequencing electrophoretograms of family members: proband A, his parents, and two brothers. A hemizygous missense mutation in IL2RG c.251A>C, p.Asn84Thr was shown in the proband. Both parents and two brothers were wild type.

Proband A represented the third male child of healthy, nonconsanguineous parents. Both of his brothers were healthy, and no presence of IEI in the family history was observed (Figure 1). The proband was primarily hospitalized at the age of three months because of recurrent fever, vomiting, and lethargy. Initial suspicion of hemophagocytic lymphohistiocytosis was excluded by cytomorphology. Peripheral blood flow cytometry revealed the complete absence of B cells (0%), almost no NK cells (0.3%), and significantly decreased residual T cells (28.7%, CD4/8 ratio 0.4), which was referred to as maternofetal engraftment. Naive T cells, including recent thymic emigrants, were completely absent, resulting in the atypical X-SCID immunophenotype T-B-NK-. Furthermore, anemia, severe thrombocytopenia, and leukopenia were diagnosed through complete blood count (CBC) analysis (Table 1). Polymerase chain reaction (PCR) revealed cytomegalovirus (CMV) infection (3.54 × 10^6^ copies/mL) in the blood, which led to treatment via intravenous ganciclovir. The measurement of sCD25, a soluble IL2RA component of the receptor IL2RG, revealed a decrease of 250 UI/mL (standard 710 IU/mL). Ophthalmological, chest X-ray, and neurological examinations involving brain ultrasonography yielded normal results.

**Table 1:** Summary of laboratory results. All the parameters were analyzed at the first hospitalization stage. The CBC tests were performed on the peripheral blood samples. The virus was detected via PCR. Analysis of immunoglobulins was performed on peripheral blood samples, and the levels represented soluble molecules. CBC, complete blood count.

Parameter	Proband A	Proband B	Normal range
Complete blood count results			
White blood cells (x10^9^/L)	0.5	15.21	4-11
Neutrophils (%)	61.6	10.2	38-30
Lymphocytes (%)	27.3	66.9	18-50
Monocyte (%)	9	8.2	2.0-10.0
Red blood cells (x10^12^/L)	2.21	3.85	3.12-7.3
Hemoglobin (g/dL)	5.7	10.4	14-17
Hematocrit (%)	0.17	0.31	41.5-50.4
Mean cell volume (fL)	78	79	80-96
Mean corpuscular hemoglobin (pg)	26	26.6	27.5-33.2
Mean corpuscular hemoglobin concentration (g/dL)	332	341	33.4-35.5
Platelets (x10^9^/L)	47	715	150-450
PCR results			
Cytomegalovirus (copies/mL)	3.54 x 10^6^	8.9 x 10^3^	--
Cytomegalovirus	Positive	Negative	--
Epstein-Barr virus	Negative	Negative	--
Human herpesvirus 6	Negative	Negative	--
Parvovirus B19	Negative	Negative	--
Adenovirus	Negative	Negative	--
Immunoglobulins			
IgG (g/L)	7.39	0.96	6.58-18.37
IgM (g/L)	<0.13	0.24	0.4-2.5
IgA (g/L)	0.13	0.09	0.8-3.5
IgE (ug/L)		1114.7	

Genetic analysis was performed at the age of five months by whole exome sequencing (WES) approach on DNA isolated from a peripheral blood sample and revealed c.251A>C, p.Asn84Thr [NM_000206.3] DNA variant in the *IL2RG *gene. This DNA variant was subsequently confirmed by Sanger sequencing to verify the presence of the identified mutation within the proband’s family. At the age of seven months, due to the suspected X-SCID causal genotype, the patient underwent allogeneic stem cell transplantation, despite active CMV infection, and the HLA-identical sibling without the *IL2RG *DNA variant was used as a donor. On the 21st day after transplantation, successful graft acceptance was observed, and the proband's clinical condition significantly improved.

Proband B was born to healthy, nonconsanguineous parents from the first physiological pregnancy, and complications were observed during parturition. Proband has one healthy younger brother. The first hospitalization occurred at the age of eight months because of generalized impetiginized dermatitis with secondary infection by *Staphylococcus* spp. and *Candida* spp. detected at eczema sites. The laboratory results of the two probands are summarized in Table 1. Immunology tests revealed an increased level of sIgE with several approved food allergies and a decreased level of IgG, leading to the initiation of chronic replacement therapy with intravenous immunoglobulin (IVIg). After IVIg therapy, the skin condition showed mild regression. Blood flow cytometry analysis revealed an atypical T+B+NK+ immunophenotype, indicating disruption of B-cell maturation, lymphocytosis (absolute lymphocyte count exceeding 4 × 10^9^/L), a predominance of naive T cells, and a normal count of NK cells. The presence of B cells in the phenotype excludes the diagnosis of Bruton or X-linked agammaglobulinemia (XLA). The control parameters of cellular immunity confirmed the T+B+NK+ immunophenotype with impaired T and B maturation but a normal number of NK cells. After nearly a year of substitution therapy, the IgG value reached 4.14 g/L, and the skin conditions stabilized, leading to a switch from intravenous IgG to fSCIg (recombinant human hyaluronidase-facilitated subcutaneous immunoglobulin). Ophthalmological, cardiological, neurological, and chest X-ray examinations revealed normal results. At the age of 19 months, genetic analysis by the WES approach, on DNA isolated from peripheral blood samples, revealed a DNA variant in the *IL2RG*, namely, c.638T>C, p.Val213Ala [NM_000206.3]. The proband was diagnosed with milder X-SCID, but not severe X-CID. The dominant manifestation of X-CID is immune dysregulation associated with polyvalent food allergy with severe skin manifestations imitating atopic dermatitis [8]. The successful initiation of IVIg/fSCIg replacement therapy and diet restrictions minimized skin manifestations, and the IgG values shifted to within the normal ranges. Owing to the patient’s improvement and mild phenotype, urgent transplantation of hematopoietic stem cells was not suggested.

## Discussion

In proband A with the WES approach, the infant was determined to be hemizygous because of the presence of potentially causal DNA variants in the *IL2RG *gene, specifically c.251A>C, p.Asn84Thr, located in exon 2. The DNA variant was automatically classified as a VUS (variant of uncertain significance) by the ACMG (American College of Medical Genetics and Genomics) criteria via the Sophia DDM, VarSome, and Franklin tools, with both amino acids representing hydrophilic and polar types. The complex results of the in silico prediction analysis are shown in Table 2.

**Table 2:** Results of in silico analysis of the identified DNA variants. SIFT: Index 1 represents pathogenic prediction, GERP++ scores range from -12.3 to 6.17; the higher scores indicate the higher evolution conservation, InterVar interpretation based on the ACMG/AMP 2015 guideline criteria. VUS, variant of uncertain significance; ACMG, American College of Medical Genetics and Genomics; AMP, Association for Molecular Pathology.

Gene	Proband A	Proband B
IL2RG	c.251A>C, p.Asn84Thr	c.638T>C, p.Val213Ala
Prediction software	Prediction results	
Mutation Taster	Disease-causing (1)	Disease-causing (1)
SIFT (Sophia)	1	0.872
PolyPhen2	Probably damaging (0.985)	Probably damaging (0.992)
GERP++	5.43	4.88
InterVar	VUS (PM1, PM2, PP3)	VUS (PM1, PM2)
AGVGD	C65 (most likely pathogenic)	C55 (likely pathogenic)
VarSome	VUS, with minor pathogenic elements PM5 moderate, PP3 moderate, PM2 supporting	VUS, PM1 moderate, PM2 supporting
Franklin	VUS, with minor pathogenic elements PM2 supporting, PP3 moderate	VUS, PM2 moderate
ClinVar	No data available	No data available

The proband’s atypical immunophenotype was determined to be T-B-NK-, in contrast with the typically reported immunophenotype associated with IL2RG defects, which is characterized as T-B+NK- [9]. T-B-NK- cells commonly exhibit *ADA *gene mutations (ADA-deficient SCID) and reticular dysgenesis related to *AK2 *gene mutations [10]. No single-nucleotide variants (SNVs) or copy number variants (CNVs) in the *ADA *or *AK2 *genes were detected in the WES analysis data, while false negativity was not expected because minimal coverage criteria were met (100% coverage at minimum 25 depth). Although immunophenotypes that differ from those of common T-B+NK- have been reported with DNA variants in *IL2RG*, e.g., T-B+NK+ [9,11] or TlowB+NK+ [12], we were not able to fully interpret and explain the absence of B cells. Even though both DNA variants represent missense mutations, they lead to different immunophenotypes in the presence of T/B/NK cells. The first one, p.Asn84Thr, leads to the T-B-NK- phenotype, while the second one, p.Val213Ala, to the T+B+NK+ phenotype. The difference may be influenced by the factor of localization of mutation in the protein; while the first one disrupts the glycosylation site, the second one only modifies one amino acid in the functional domain and thus can represent a hypomorphic variant.

In contrast with automatic VUS classification, the variant is located at a glycosylation site in the conserved extracellular protein domain within codons p.T23-A262. The glycosylation sites in the IL2RG protein serve as posttranslational modification regions targeted for saccharide bonding, which is important for correct cellular recognition, adhesion, protein stability, folding, and the regulation of biological activities [13]. Changes at the glycosylation sites in the IL2RG protein could lead to misfolding, instability, impaired cellular interactions, and disrupted signaling. 

We define that the detected *IL2RG* DNA variant c.251A>C is of de novo origin, while not detected in the parents (Figure 1).

Moreover, Bandari et al. described the adjacent DNA variant c.252C>A in the same codon (84) and classified it as pathogenic, although the Varsome tool prediction based on the ACMG criteria was likely pathogenic, and the Franklin tool predicted it as VUS based on the ACMG criteria [14]. Furthermore, we manually appended the PS2 (concerning the de novo status) and PS1 (concerning known pathogenic variants in the same codon) criteria in VarSome and Franklin tools and subsequently reclassified the variants into the likely pathogenic class. This reclassification was also supported by the AGVGD prediction (Table 2). Based on this information, we conclude that the identified variant, located in a region critical for the correct function of the protein, was likely pathogenic.

In proband B, using WES analysis, we identified a hemizygous missense DNA variant, c.638T>C, p.Val213Ala, in the *IL2RG *gene. The variant has been classified by an automatic algorithm as a VUS in VarSome and Franklin, and the prediction results are summarized in Table 2. Both substituted amino acids represent hydrophobic and nonpolar types, indicating no critical substitution.

The proband has shown an atypical T+B+NK+ immunophenotype, while the NK+ immunophenotype was previously observed mainly in patients with mutations in exon 3 [15,16], exon 5 [7], and exon 7 of the *IL2RG *gene [16], which is correlated with the location of our variant. Fuchs et al. described a patient with a similar T+NK+ immunophenotype and identified a missense variant, c.664C>T, p.Arg222Cys, in exon 5 of the *IL2RG *gene [7]. Moreover, Lim et al. reported that approximately 10% of the *IL2RG *pathogenic variants associated with a milder X-CID phenotype presented a “leaky” form of immunodeficiency [17].

The variant c.638T>C is located in exon 5 within a region encompassing two functional protein domains, the fibronectin type III domain and the disulfide bond domain. The fibronectin type III domain plays a crucial role in cell adhesion, signaling, and receptor-ligand interactions. This domain contributes to the activity and structural integrity of the IL2RG receptor, whereas the second functional disulfide bond domain contributes to the stability of the protein [18]. According to the literature, 362 pathogenic *IL2RG *variants have been reported, the majority of which, accounting for 29%, are located in exon 5, and 38% of them represent missense variants as the dominant mutation type [16], which is in agreement with our identified variant c.638T>C. Touvinen et al. reported two nonrelative probands with a novel *IL2RG *missense variant, c.172C>T, p.Pro58Ser, which affects the impaired expression of the IL2R complex. Both patients presented with a nonsevere X-CID phenotype with a T+B+NK+ immunophenotype and suffered from recurrent respiratory tract infections, bronchiectasis, and reactive arthritis [19]. Owing to hypomorphic mutations, genetic reversions in early progenitor cells, or maternal T/NK cell engraftment, these “leaky” phenotypes may display preserved and/or partially functional T/NK cell subsets [20].

**Figure 2:**
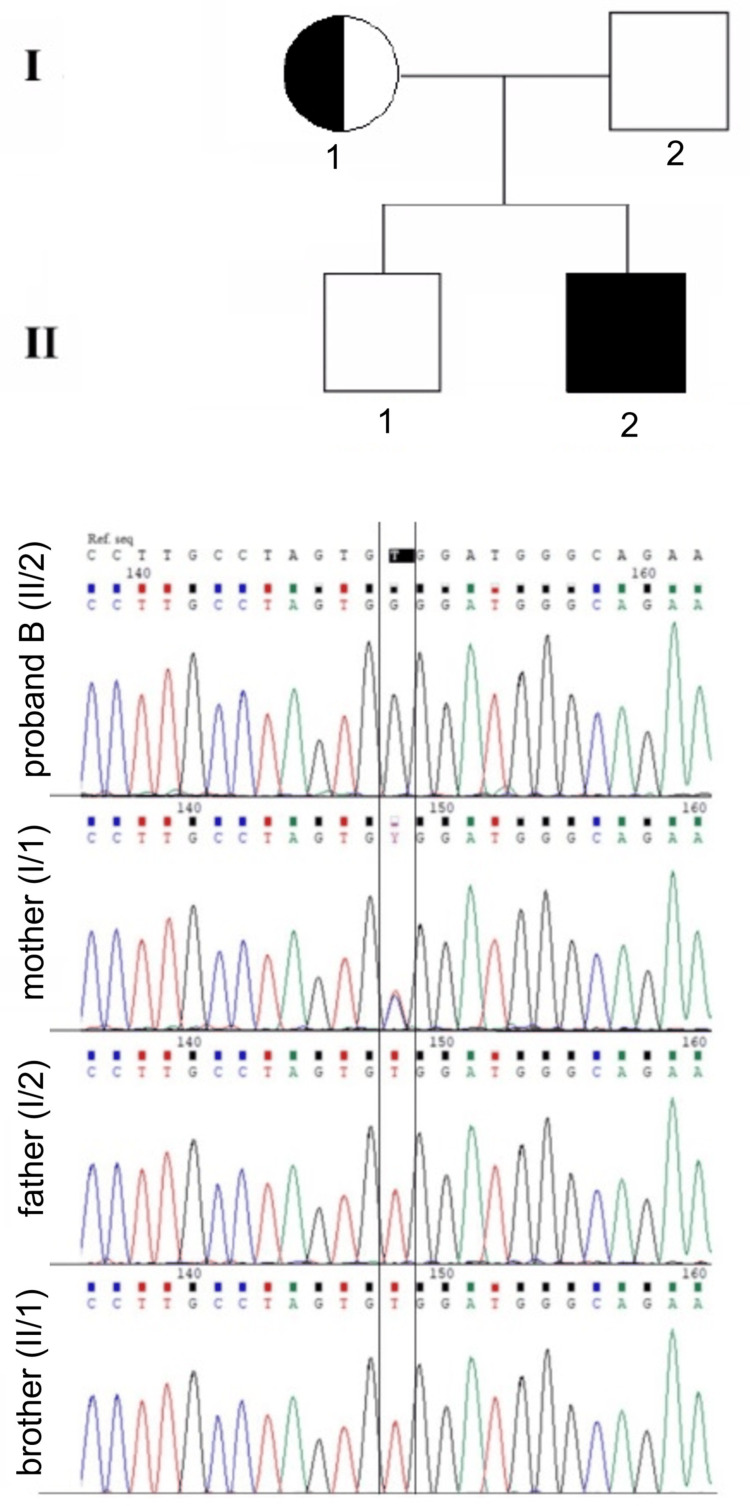
Pedigree and Sanger sequencing electrophoretograms of family members: proband B, his parents, and brother. A hemizygous missense mutation in IL2RG c.638T>C, p.Val213Ala was shown in the proband. Mother has shown heterozygous missense mutation, and father and brother were wild type.

Segregation analysis of the proband's family revealed the heterozygous presence of the variant in the proband's mother (Figure 2); thus, she represents an asymptomatic carrier, which is in concordance with the X recessive inheritance pattern.

In the VarSome database, a DNA variant two bases upstream of c.638T>C was reported, representing a frameshift variant c.640del in codon 214, which is predicted to be pathogenic, and in the database Franklin, it is considered likely pathogenic. We further classified the variant by adding the PP1 ACMG criterion (concerning cosegregation of the variant with disease) in VarSome and Franklin, leading to the reclassification of the variant as VUS with minor pathogenic elements. Additionally, the manual AGVGD prediction shifted the variant into the higher pathogenicity class (Table 2).

Owing to the moderate pathogenic in silico predictions of the variant c.638T>C, the milder phenotype of the patient, and the lack of observed associations because of the rarity of the variant, we can conclude that the variant is likely pathogenic but is associated with mild penetrance and therefore leads to a milder X-CID phenotype.

Even though both DNA variants represent missense mutations, they lead to different immunophenotypes in the presence of T/B/NK cells. The first one, p.Asn84Thr, leads to the T-B-NK- phenotype, while the second one, p.Val213Ala, to the T+B+NK+ phenotype. The difference may be influenced by the factor of localization of mutation in the protein; while the first one disrupts the glycosylation site, the second one only modifies one amino acid in the functional domain and thus can represent a hypomorphic variant.

However, the limitation of our study rests on the lack of a functional study to directly confirm the pathogenicity of the identified *IL2RG *variants, which should be performed in the future. Functional assay validation is important to further understand how these mutations affect immune cell development and the overall immune response and modulate the immunophenotype of patients. 

## Conclusions

In summary, we identified and characterized two novel and potentially causal DNA variants in the *IL2RG *gene in a hemizygous state in two nonrelative X-SCID patients in the Slovak population. These variants were initially automatically evaluated as VUS, mainly because of the lack of information from population databases and because of the type of amino acid substitution. However, using segregation analysis data, different genomic tools and databases, additional biological and prediction data, and summarization of clinical data, we further evaluated and reclassified the variant c.251A>C, p.Asn84Thr as likely pathogenic and the variant c.638T>C, p.Val213Ala as likely pathogenic with mild penetrance. Publicly available data also suggest that both DNA variants represent novel, recently undescribed DNA variants worldwide, identified especially in X-SCID patients from the Slovak population.

Identifying novel *IL2RG *variants has significant clinical implications for the diagnosis, treatment, and genetic counseling of patients with X-SCID. These findings can aid in early and accurate diagnosis, particularly for cases where standard immunophenotyping may not provide clear results, as seen in proband B with a milder phenotype. By identifying these specific variants, clinicians can confirm the diagnosis and better stratify the severity of the disease, which is crucial for tailoring treatment plans, such as determining the need for hematopoietic stem cell transplantation or gene therapy. Furthermore, these findings can improve genetic counseling for affected families. Knowing the specific variants in the *IL2RG *gene allows for better risk assessment and carrier testing in family members. 

For the further validation of the findings of our study, functional assays should be performed to experimentally assess the impact of the identified *IL2RG* variants on protein function. These studies could include in vitroassays to evaluate protein expression, receptor activity, and signaling affected by these mutations. Additionally, population-based studies could help explore the prevalence and clinical significance of these variants across different populations, which may contribute to refining diagnostic strategies and therapeutic approaches.
